# Cataloguing experimentally confirmed 80.7 kb-long *ACKR1* haplotypes from the 1000 Genomes Project database

**DOI:** 10.1186/s12859-021-04169-6

**Published:** 2021-05-26

**Authors:** Kshitij Srivastava, Anne-Sophie Fratzscher, Bo Lan, Willy Albert Flegel

**Affiliations:** Laboratory Services Section, Department of Transfusion Medicine, NIH Clinical Center, National Institutes of Health, Bethesda, MD 20892 USA

## Abstract

**Background:**

Clinically effective and safe genotyping relies on correct reference sequences, often represented by haplotypes. The 1000 Genomes Project recorded individual genotypes across 26 different populations and, using computerized genotype phasing, reported haplotype data. In contrast, we identified long reference sequences by analyzing the homozygous genomic regions in this online database, a concept that has rarely been reported since next generation sequencing data became available.

**Study design and methods:**

Phased genotype data for a 80.6 kb region of chromosome 1 was downloaded for all 2,504 unrelated individuals of the 1000 Genome Project Phase 3 cohort. The data was centered on the *ACKR1* gene and bordered by the *CADM3* and *FCER1A* genes. Individuals with heterozygosity at a single site or with complete homozygosity allowed unambiguous assignment of an *ACKR1* haplotype. A computer algorithm was developed for extracting these haplotypes from the 1000 Genome Project in an automated fashion. A manual analysis validated the data extracted by the algorithm.

**Results:**

We confirmed 902 *ACKR1* haplotypes of varying lengths, the longest at 80,584 nucleotides and shortest at 1,901 nucleotides. The combined length of haplotype sequences comprised 19,895,388 nucleotides with a median of 16,014 nucleotides. Based on our approach, all haplotypes can be considered experimentally confirmed and not affected by the known errors of computerized genotype phasing.

**Conclusions:**

Tracts of homozygosity can provide definitive reference sequences for any gene. They are particularly useful when observed in unrelated individuals of large scale sequence databases. As a proof of principle, we explored the 1000 Genomes Project database for *ACKR1* gene data and mined long haplotypes. These haplotypes are useful for high throughput analysis with next generation sequencing. Our approach is scalable, using automated bioinformatics tools, and can be applied to any gene.

**Supplementary Information:**

The online version contains supplementary material available at 10.1186/s12859-021-04169-6.

## Introduction

Data generated by next generation sequencing (NGS) are often utilized in the emerging fields of precision and personalized medicine. This massively parallel processing chemistry can identify genetic factors that predict treatment and response to therapies. Reference nucleotide sequences are critical for analyzing NGS data, as exemplified by routine clinical diagnosis for HLA antigens [1].

Genotype phasing is the process to determine if genetic variants, often single nucleotide variations, called SNVs, belong to 2 separate chromosomes (*in trans*). If SNVs are located on the same chromosome (*in cis*), they constitute a haplotype or an allele. Genotype phasing has often been inferred using computational methods [2, 3], which are prone to certain types of error [4]. These errors are encountered in samples harboring novel variants, low frequency or rare variants, and structural variants [5]. Almost all of these errors can be precluded by laboratory based methods, such as sequencing the genomes of both parents and sibling offspring [6], physical separation of homologous chromosomes in diploid cells [7, 8], sequencing in sperm cells [9], allele specific PCR [10], single DNA molecule dilution [11] and single molecule sequencing chemistry [12, 13]. These laboratory based methods are, however, labor-intensive and time consuming, and thus infrequently applied in clinical diagnostics.

The human genome contains many regions that are known as long contiguous stretches of homozygosity (LCSH) [14,15]. Their presence in unrelated individuals across different populations is attributed to a lower average recombination rate in these regions of the human genome [14].

The human atypical chemokine receptor 1 gene (*ACKR1*, MIM #613,665) [16] encodes a multi-pass trans-membrane glycoprotein. It is a receptor for pro-inflammatory cytokines [17] and malaria *Plasmodium* parasites (*P. vivax* and *P. knowlesi*) [18]. The ACKR1 glycoprotein carries the five antigens of the Duffy blood group system (Fy) [19, 20]. Recent sequencing studies in the *ACKR1* gene have identified approximately 30 haplotypes, albeit at limited lengths of 2.1 kb [21], 2.5 kb [22], 5.2 kb [23], and 5.6 kb [24], respectively. We previously applied these *ACKR1* haplotypes to predict the Duffy phenotype in Neanderthal samples [21]. Later, high-coverage genome sequences of Neanderthals were established [25–27], which confirmed our prediction [21]. A recent similar comparative study, involving long genomic segments, identified a 50 kb segment in humans, which was inherited from Neanderthals and represented a genetic risk factor in SARS-CoV-2 infection [28].

The 1000 Genomes Project (1000GP) provides a comprehensive database of genotypes and haplotypes in 2,504 unrelated individuals across 26 populations worldwide [29, 30]. As a proof of principle using data from the 1000GP for the *ACKR1* gene, we establish a list of 902 haplotypes, some more than 80 kb long. Our scalable approach can be applied to any gene in any population.

## Materials and methods

### Algorithm workflow

**Fig. 1:**
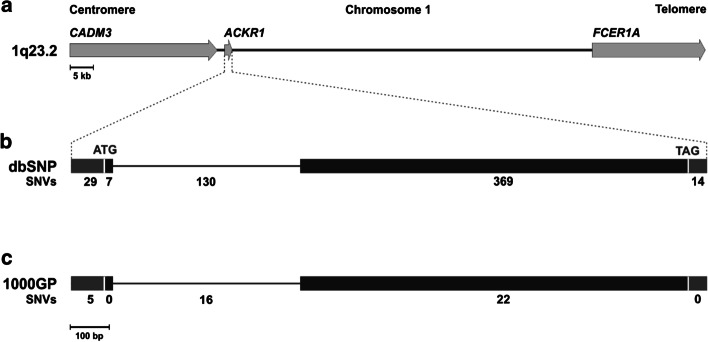
Schematic representation of chromosome 1 region analyzed. The *ACKR1* gene is bordered by the 2 genes *CADM3* in centromeric and *FCER1A* in telomeric direction at chromosomal position 1q23.2 (**a**). The structure of the *ACKR1* gene **(b)** comprises 2 exons (closed boxes) and include the coding sequence (CDS,  black) and the 5’- and 3’-untranslated region (UTR, grey). The intron 1 joins the 2 exons (black line). The number of SNVs observed in the for the dbSNP **(b)** and 1000GP databases **(c)** are shown for the 5’-UTR, CDS, intron, CDS and 3’-UTR

A Python algorithm was developed (Supplementary Information, File S1) to download and analyze genotype data for 80.6 kb region of chromosome 1 (between positions NC_000001.11: 159,203,314–159,283,887) flanked between 2 genes, *CADM3* and *FCER1A*, and encompassing the *ACKR1* gene (Fig. 1) for all 2,504 unrelated individuals of the final release 1000GP panel (Phase 3; GRCh38) using Bcftools [31]. The SNV data was downloaded from the dbSNP database [32]. Individual sequences with heterozygosity at a single site or with complete homozygosity were automatically extracted as an unambiguous *ACKR1* haplotype that can be considered experimentally confirmed, which applied a time-proven concept [4]. The algorithm outputs three files: a sequence file containing the distinct haplotypes, a meta-data file containing information about the population in which the haplotypes are found, and a folder containing graphical representations of the population distribution of the distinct haplotypes.

### Validation

Phased haplotype data for 80.6 kb region of chromosome 1 (between positions NC_000001.11: 159,203,314–159,283,887) was manually downloaded for all 2535 individuals of the 1000GP panel (Phase 3; GRCh37) from the 1000 Genomes browser. After removing 31 related individuals, haplotype data from 2504 unrelated individuals was imported into Microsoft Excel. Individuals with heterozygosity at a single site or with complete homozygosity in the 1,626 nucleotide-long *ACKR1* gene (NG_011626.3; NC_000001.11:159,204,875–159,206,500) allowed unambiguous assignment of an *ACKR1* haplotype. These unambiguous *ACKR1* haplotypes were further analyzed individually using Excel spreadsheets, and their sequences were extended in both 5'- and 3'-directions until a heterozygous SNV was encountered. The region between 2 SNVs was catalogued as a haplotype and compared with the previous automated results. The manual analysis was performed and thus a validation dataset generated before the Python algorithm was developed.

### Neanderthal genome

The published DNA sequence of the Neanderthal genome (Chagyrskaya, Altai, and Vindija 33.19, http://cdna.eva.mpg.de/neandertal/) [25–27] was analyzed (Integrative genomics viewer version 2.3.20) [33] and aligned to the human genome (NCBI Build GRCh38/hg38). We searched for the longest match, if any, with the haplotypes in the 1000GP.

## Results

Using the 1000GP database and a Python algorithm, we extracted and catalogued long haplotypes that encompassed the *ACKR1* gene and were flanked between 2 SNVs (Fig. 1). Among 2,504 individuals included in the 1000GP database, 1,520 individuals were homozygous for the 1,626 nucleotide-long *ACKR1* gene or heterozygous with only 1 SNV. The *ACKR1* sequences for these individuals were further analyzed both upstream and downstream of *ACKR1* gene until SNVs were encountered. The extension in both directions allowed us to identify long *ACKR1* haplotypes that can be considered experimentally verified. The results obtained with our computational approach were validated by a manual method, performed in a blinded fashion.

### ACKR1 and SNVs

For the *ACKR1* gene (Fig. 1), the dbSNP database [32] lists 549 SNVs spread over 1,626 nucleotides (Fig. 1b). We encountered, however, only 43 SNVs of the *ACKR1* gene in the 1000GP database (Fig. 1c) out of the 549 known SNVs.

### *ACKR1* haplotypes

**Fig. 2:**
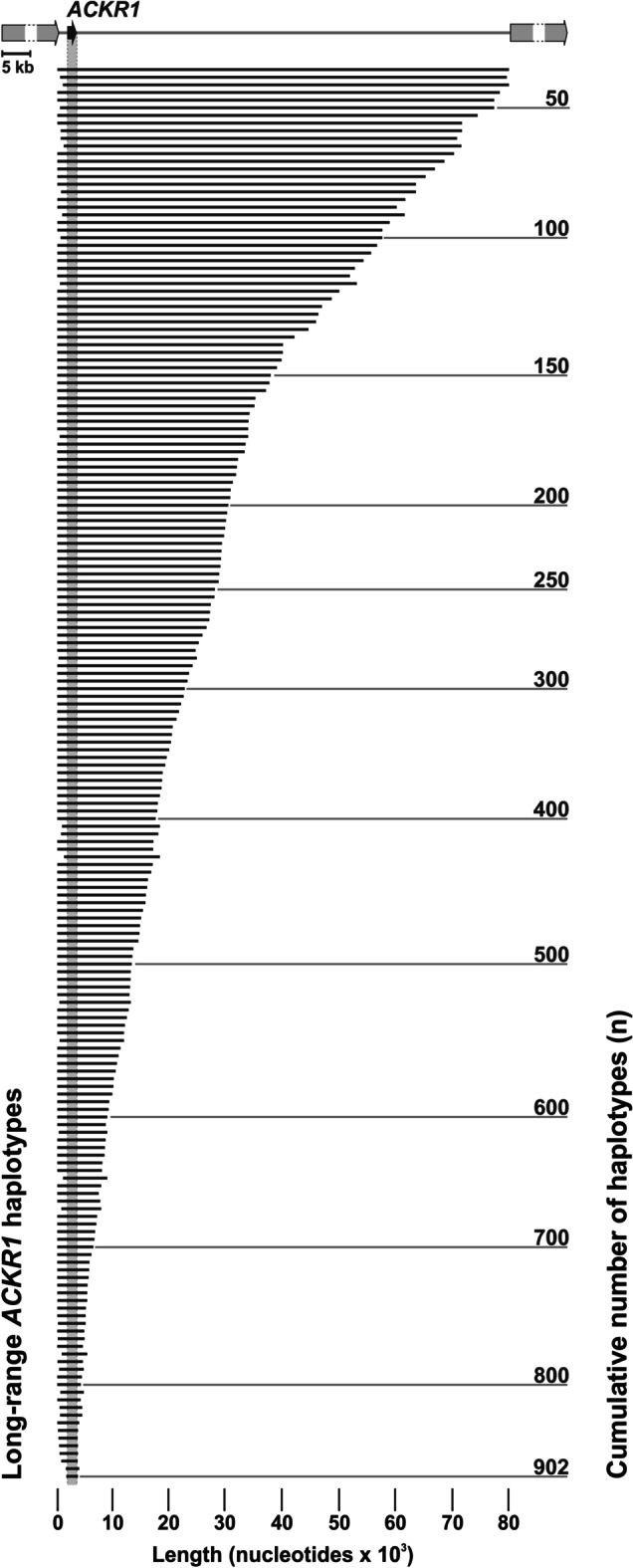
*ACKR1* haplotypes observed in the 1000GP. A total of 902 unique haplotypes were observed and sorted according to their length (bars). All haplotypes comprise the *ACKR1* gene (shaded column), their positions in the *ACKR1* gene locus (top, see Fig. 1) is indicated. The cumulative number is listed (right). Haplotypes of similar lengths are grouped together (for exact lengths see Supplementary Information, Excel files S1 and S2)

**Fig. 3:**
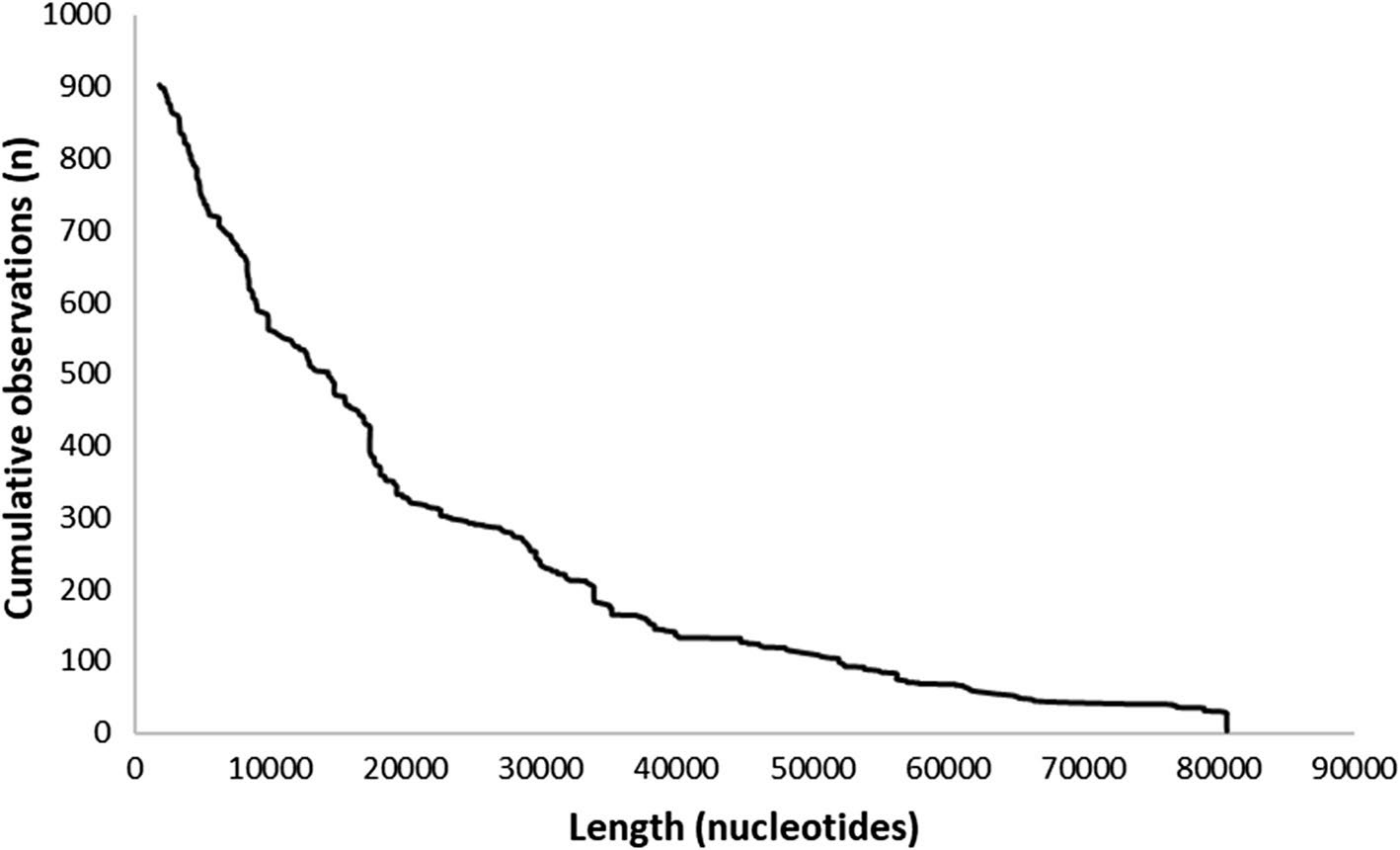
Correlation between length and observations of *ACKR1* haplotypes. The length of the *ACKR1* haplotypes (x-axis) observed in the 1000GP was inversely proportional to the number of observations (y-axis)

We identified 31 distinct haplotypes with ≥ 10 observations (Table 1). They ranged in length from 2,383 nucleotides to 17,739 nucleotides. A total of 902 haplotypes were observed, ranging in length from 1,901 nucleotides to 80,584 nucleotides, some extending into the adjacent *CADM3* and *FCER1A* genes (Fig. 2). The combined length of haplotype sequences comprised 19,895,388 nucleotides with a median of 16,014 nucleotides (Quartile 1 – Quartile 3: 7,588 – 30,729 nucleotides; Interquartile Range: 23,141 nucleotides). The length of the haplotypes was inversely proportional to the number of observations (Fig. 3). Most of the common haplotypes (70.13%) were small (< 10 kb; Table 2) and ranged in length between 1,901 to 9,927 nucleotides. The most common *ACKR1* allele observed was the Duffy-null allele (FY*02 N.01) followed by FY*A (FY*01) and FY*B (FY*02), respectively (Table 3). For each of these 3 common *ACKR1* alleles, we were able to identify reference sequences longer than 80 kb (Table 3).

**Table 1:** Experimentally confirmed *ACKR1* haplotypes with ≥ 10 observations in the 1000GP database*

Haplotype	Length (nucleotides)	Observations (n)					Total
		Super-population†					
		AFR	AMR	EAS	SAS	EUR	
01	3385	1	39	149	83	28	300
02	3386	1	37	149	76	21	284
03	5168	161	1	0	0	0	162
04	5168	160	1	0	0	0	161
05	2483	107	1	0	0	0	108
06	2483	107	1	0	0	0	108
07	4871	0	6	42	0	1	49
08	4871	0	5	42	0	1	48
09	4376	0	0	36	0	0	36
10	4376	0	0	35	0	0	35
11	6276	27	0	0	0	0	27
12	6276	25	0	0	0	0	25
13	9091	20	1	0	0	0	21
14	17,406	0	4	1	15	1	21
15	14,785	0	4	4	10	2	20
16	2383	0	5	0	3	11	19
17	17,405	19	0	0	0	0	19
18	2383	0	5	0	3	11	19
19	17,739	16	1	0	0	0	17
20	3385	0	2	0	7	7	16
21	2620	0	7	0	0	9	16
22	2620	0	7	0	0	9	16
23	6310	0	0	15	0	0	15
24	6310	0	0	15	0	0	15
25	4869	0	3	10	2	0	15
26	2706	0	1	1	4	8	14
27	2706	0	1	1	4	8	14
28	9092	11	1	0	0	0	12
29	4644	0	2	0	4	5	11
30	4643	11	0	0	0	0	11
31	4644	0	2	0	4	4	10

^*^Besides these haplotypes with ≥ 10 observations, a total of 902 *ACKR1* haplotypes were confirmed (see Fig. 2), 871 of which had < 10 observations each

^†^Super-population as defined by the 1000GP [29,2: Table S1)

**Table 2:** *ACKR1* haplotypes and length distribution in the 1000GP database among 1520 individuals

Length range (nucleotides)	*ACKR1* haplotypes	
	Observations* (n)	Frequency (%)
< 10,000	2,132	70.13
10,000 – 19,999	468	15.39
20,000 – 29,999	128	4.21
30,000 – 39,999	132	4.34
40,000 – 49,999	34	1.12
50,000 – 59,999	52	1.71
60,000 – 69,999	26	0.86
70,000 – 79,999	16	0.53
≥ 80,000	52	1.71
Total	3,040	100

^*^Among 2,504 individuals included in the 1000GP database, 1,520 individuals (3,040 chromosomes) were homozygous for the 1,626 nucleotide-long *ACKR1* gene or heterozygous with only 1 SNV

**Table 3:** Length distribution of the 3 common *ACKR1* alleles observed in the 1000GP

ISBT allele	Haplotype*	Observations†	Length range	Mean ± standard deviation	Median
FY*01	TGCCGCGCCGCGGGC	389	2241—80,576	24,628 ± 22,298	16,874
FY*02	TACCGCGCCGCGGGC	166	1901—80,576	24,851 ± 23,186	13,779
FY*02 N.01	CACCGCGCCGCGGGC	344	1977—80,584	18,482 ± 15,755	15,125
Total		899	1901—80,584	22,098 ± 19,903	16,315

^*^The nucleotides at the 15 SNV positions are shown in 5' to 3' orientation (Additional file 5:Table S4)

^†^Variant positions in the intron and synonymous variants in exons are ignored. Rare Fy(a+^w^) and Fy(b+^w^) encoding alleles are also ignored (see Additional file 5:Table S4)

### *ACKR1* alleles in the Neanderthal samples

The 3 Neanderthal samples were *GATA box* negative (-67 T) and represented the ancestral FY*B allele (Table 4). None of the 3 Neanderthal *ACKR1* sequences (Chagyrskaya, Altai, and Vindija 33.19) fully matched any of the 902 haplotypes. The 2 haplotypes closest to the Neanderthal sequences had 1 mismatch in the *GATA box* (Table 4).

**Table 4:** *ACKR1* alleles in the 1000GP and 3 Neanderthal samples

Haplotype	Observations			Nucleotides position*				Length (base pairs)
	Species	Population†	n	c.-67 T > C	c.21 + 115 T > C	c.21 + 235 T > C	c.125G > A	
NG_011626.3‡	*H. sapiens*	NA	NA	T	T	T	G	1,626
HAP897	*H. sapiens*	ACB	1	C	C	T	A	2,032
HAP899	*H. sapiens*	LWK	1	C	C	T	A	1,978
Chagyrskaya	*H. neanderthalensis*	NA	1	T	C	T	A	NA
Altai	*H. neanderthalensis*	NA	1	T	C	T	A	NA
Vindija	*H. neanderthalensis*	NA	1	T	C	Y	A	NA

^*^Nucleotide positions are shown according to the human reference sequence (NG_011626.3) and defined using the first nucleotide of the coding sequence (CDS) of the NM_002036.2 isoform as nucleotide position 1. Only variant positions with respect to the 2,032 nucleotides of the HAP897 are listed

^†^ACB = African Caribbeans in Barbados; LWK = Luhya in Webuye, Kenya

^‡^*ACKR1* reference allele per ISBT [95]

NA, not applicable; Y = T or C

## Discussion

In the current study, we identified 902 experimentally confirmed reference haplotypes for the *ACKR1* gene, using only publicly available data from the large scale 1000GP study database. Our approach is easily scalable. It can be applied to similar databases, including the UK10K Consortium [34], the African Genome Variation Project [35] and the upcoming All of Us Research Program [36]. For proof of principle, we demonstrated the application using a Python algorithm for one gene. The approach can, however, define reference sequences for any segment of the genome, with genes or without.

We showed that reference sequences can be obtained from databases and verified without ambiguity at lengths exceeding 80 kb. Such reference sequences can be catalogued inexpensively for use in clinical diagnostics. The catalogue comprised the set of the longest unique haplotypes that can be distinguished by the gene’s nucleotide sequence. In clinical diagnostics with molecular-based assays, common and well documented (CWD) [37] reference haplotypes are routinely applied, for example in HLA typing [1]. Exact matching at the haplotype level improves survival following bone marrow transplantation [38] and reduces alloimmunization in chronically transfused patients [39–41]. A limited number of common haplotypes represented the majority in the population [42], and identifying haplotypes from databases is an economical way to obtain such reference sequences.

Apart from clinical diagnostics, long-range haplotypes are also useful to understand the influence of environment on positive selection of genes in human populations [43], for association mapping of genes that contribute to disease and other phenotypes [44], for correlating the geographical distribution of haplotypes with endemicity of disease [45], for identifying evolutionarily conserved elements and regulatory elements [46], and for improving the reliability of genotype imputation [47]. Long haplotypes identified by using SNV data from high-density oligonucleotide arrays and the International HapMap Project [48] have been shown to be population dependent and can provide important insights into human evolutionary history [49]. These studies may also identify regions of positive selection with important roles in human health and disease [50].

Next generation sequencing is increasingly used for blood group genes [51–78]. In contrast to HLA [79], most blood group genes lack well documented long reference sequences associated with them [80]. Hence, a comprehensive reference database for blood group genes will facilitate blood group genotyping by NGS. The Erythrogene database [59] contains the complete coding region sequence of many different blood group alleles obtained from the 1000GP. However, it lacks information for sequence variants in the non-coding regions, such as promoter, splice sites and long intronic regions, which can also affect the expression of antigens and helps to ascertain the allele and its coding sequence [81–84].

A large number of haplotypes were more than 50 kb long with some extending at least to 80.5 kb in length (Fig. 2). Our observations are consistent with previous reports suggesting that most of the human genome is contained in blocks of a few kb to more than 100 kb [85, 86]. However, most of the *ACKR1* haplotypes in the 1000GP were small and concentrated closely around the *ACKR1* gene. The number of haplotypes decreased as their length increased and extended into the intergenic regions (Fig. 3). This is explained because most of the variants in the dbSNP database resides in the intergenic regions [87].

Our 2 haplotypes HAP897 and HAP899 (Additional file 4:Table S3), observed once each in African populations, were closest to the 3 Neanderthal samples. Both haplotypes carried the *GATA box* mutation (c.-67C), which all Neanderthal samples lacked (c.-67T). Individuals homozygous for the *GATA box* mutation (c.-67C) do not express the Duffy glycoprotein on the red cell surface [81] making them resistant to invasion by the malarial parasite *P. vivax* [88–90]. This similarity in alleles, discrepant at nucleotide position c.-67 only, was consistent with the fact that the *GATA box* mutation (c.-67C) started to spread in Africa only around 30,000 years ago [91], while the 3 Neanderthals Vindija, Altai and Chagyrskaya are 50,000, 120,000 and 50,000 years old, respectively [25–27].

In clinical diagnostics for patients, long-range haplotypes harboring novel or rare SNVs can only be detected when the haplotype is sequenced at full-length [92]. Using Sanger sequencing, we have previously characterized the *ERMAP* [93], *ICAM4* [94], and *ACKR1* [23] blood group genes at the haplotype level and identified prevalent long-range reference alleles, a time consuming and low throughput approach. We showed in this study how long contiguous stretches of homozygosity (LCSH) can serve to generate a database of long haplotypes, as defined by full length nucleotide sequences rather than the concatenation of known SNVs. Relying on SNV data would miss patients carrying novel or rare alleles with possible clinical relevance, which are not identical to the reference sequences. Features of the 1000GP allowed us to catalogue these extended nucleotide sequences with population specific frequencies. Our approach will enable the positive identification of patients carrying these reference sequences.

We plan to extend this approach to all blood group systems recognized by the International Society of Blood Transfusion (ISBT) [95]. A tool under development will allow researchers the customized online extraction of long haplotypes from databases and genes or genomic regions of their choice. Eventually, our approach can be applied to any region of a chromosome. For now, the 902 *ACKR1* alleles identified through our novel approach will be useful as templates for analyzing data from NGS, thus enhancing the reliability of clinical diagnostics.

### Web Resources

1000 Genomes browser (https://www.ncbi.nlm.nih.gov/variation/tools/1000genomes/) accessed on Aug 05, 2019. ISBT (https://www.isbtweb.org/fileadmin/user_upload/Table_of_blood_group_systems_v6.0_6th_August_2019.pdf). Max Planck Institute for Evolutionary Anthropology (http://cdna.eva.mpg.de/neandertal/).

## Supplementary Information

**Additional file 1** **File S1**. Python algorithm.

**Additional file 2** **Table S1**. Populations in the 1000GP database.

**Additional file 3** **Table S2**. Sequence data for the 902 long range ACKR1 haplotypes in the 1000GP.

**Additional file 4** **Table S3**. Metadata file for the ACKR1 long range haplotypes in the 1000GP.

**Additional file 5Table S4**. Exonic SNV distribution in the 902 experimentally confirmed ACKR1 haplotypes.

## Data Availability

The datasets analyzed and generated during the current study are available as supplementary tables and at 1000 Genomes browser (https://www.ncbi.nlm.nih.gov/variation/tools/1000genomes/).
