# Supplementary material for: Cataloguing experimentally confirmed 80.7 kb-long ACKR1 haplotypes from the 1000 Genomes Project database
Source: BMC Bioinformatics. 2021 May 26;22:273. doi: 10.1186/s12859-021-04169-6 (PMC8150616; doi:10.1186/s12859-021-04169-6)
Supplement: Supplementary file 1 — Additional file 1 File S1. Python algorithm. [file 12859_2021_4169_MOESM1_ESM.pdf]

```
'''
FILE: main.py
PURPOSE: main entry point to application
INPUT: none
OUTPUT: none
'''
import config
import selection
import run
import os
import time

def main():
	# INSERT EMAIL HERE
	config.__EMAIL__ = "example@example.com"

	start_time = time.time()
	selection.main()
	run.main()

	print("--- %s seconds ---" % (time.time() - start_time))

if __name__ == '__main__':
	main()

'''
FILE: run.py
PURPOSE: runs computations (after data has been retrieved)
INPUT: none
OUTPUT: none
CREATED BY: Anne-Sophie Fratzscher
'''
import config
import time
import cleaner
import haplotypes
import distinct
import visualization
import popcounts
import userfile

'''
FUNCTION: computation()
PURPOSE: runs algorithm
```

```
INPUT: none
OUTPUT: none
'''
def computation():
	cleaner.main()
	haplotypes.main()
	distinct.main()
	popcounts.main()
	visualization.main()
	userfile.main()

def main():
	computation()

'''
FILE: selection.py
PURPOSE: connects to 1000GP ftp and fetches data based on range computed in fetch.py
INPUT: none
OUTPUT: error code (-1 if error)
CREATED BY: Anne-Sophie Fratzscher
'''

import config
import os
import pandas as pd
from pathlib import Path
import subprocess
import read_vcf
import fetch

def autosomes(filepath):
	config.__FILENAME__ = "1000G_chr" + (str(int(config.__CHR__)) + "_" +
							str(config.__START__) + "-" +
							str(config.__END__) + ".vcf")
	return

def createFolder(filepath):
	config.__FILEPATH__ = filepath + "/results/" + config.__GENENAME__ + "/"
	geneFolder = filepath + "/results/" + config.__GENENAME__
	#if no folder, create folder
	if not (os.path.isdir(geneFolder)):
		try:
		   Path(geneFolder).mkdir(parents=True, exist_ok=True)
		except OSError:
```

```
                print ("Creation of the directory %s failed" % geneFolder)
        return

def run_commands(*commands):
        os.system(' ; '.join(commands))
def combine():
        raw = read_vcf.read_vcf(config.__FILEPATH__+'raw_chr' + str(config.__CHR__) + "_" +
str(config.__START__) + "-"
                        + str(config.__END__) + ".vcf")
        chrom = str(config.__CHR__)

        dbSNP = pd.read_csv(config.__FILEPATH__+'dbSNP_chr'+ chrom + "_" +
str(config.__START__) + "-"
                + str(config.__END__) + ".vcf", sep='\t', header=None) # ISSUE HERE
        dbSNP.columns = ['POS', 'ID', 'REF', 'ALT']

        for i in range(0, len(raw.index)):
                # if NOT in dbSNP, dont replace (remove later)
                if not (dbSNP.loc[dbSNP['POS'] == raw.loc[i]['POS']]).empty: # check if pos match
                        row = dbSNP.loc[dbSNP['POS'] == raw.loc[i]['POS']].values[0]
                        if ((raw.iloc[i,3] == row[2]) and (raw.iloc[i,4] == row[3])): # only replace if
REF/ALT match
                                raw.iloc[i,2] = row[1] # ID
                                raw.iloc[i,3] = row[2] # REF
                                raw.iloc[i,4] = row[3] # ALT
        df = raw[raw.ID != '.'] # remove if not in dbSNP
        df.to_csv((config.__FILEPATH__ + config.__FILENAME__), sep="\t", mode='a',
index=False)

# for P3 grch38 b154
def makeCommandsP3_38_154(name, ftp, cmds, xy):
        if cmds == "":
                cmds = []
        #define ftp
        cmds.append(ftp+name)

        #define dbSNP ftp -> GRCH38 BUILD 154
        baseUrl = 'dbSNPFTP=ftp://ftp.ncbi.nih.gov/snp/redesign/latest_release/VCF/'
        refVersion = 'GCF_000001405.38.gz'
        cmds.append(baseUrl+refVersion)

        #command to get data file
        baseName =  config.__FILENAME__[len('1000G_'):]
        cmds.append('bcftools view "$ftp" -I -r ' + str(config.__CHR__) + ":"
```

```
                + str(config.__START__) + "-" + str(config.__END__)
                + " > " + config.__FILEPATH__ + "raw_" + baseName)
    cmds.append('bcftools query "$dbSNPFTP" -f "%POS\t%ID\t%REF\t%ALT\n" -r ' +
config.__CHRVERSION__
                        + ":" + str(config.__START__) + "-" + str(config.__END__) +
"> " + config.__FILEPATH__ + "dbSNP_" + baseName)

    # clean up
    cmds.append('rm ' + name + '.tbi')
    cmds.append('rm ' + refVersion + '.tbi')
    return cmds
def getDataP3_38_154(filepath):
    createFolder(filepath) # create folder if doesnt exist

    #if DONT have data, fetch
    if not Path(config.__FILEPATH__ + config.__FILENAME__).is_file():
        # if dont have raw data, fetch
        rawName = "raw_" + config.__FILENAME__[len('1000G_'):]
        if not Path(config.__FILEPATH__ + rawName).is_file():
            ftp =
"ftp=ftp://ftp.1000genomes.ebi.ac.uk/vol1/ftp/release/20130502/supporting/GRCh38_position
s/"
            vcfgzName = "ALL.chr" + str(config.__CHR__) +
"_GRCh38.genotypes.20170504.vcf.gz"
            cmd = makeCommandsP3_38_154(vcfgzName, ftp, "", "")
            run_commands(*cmd)

        #then combine raw and dbSNP
        combine()

# MAIN DECISION: picks which getData and makeCommands to use
def getData(filepath):
    autosomes(filepath)
    if config.__REFVER__ == '38':
        getDataP3_38_154(filepath)
def selectGene(filepath):
    errCode = fetch.main()
    if errCode == -1: # gene not found
        return(-1)
    config.__GENENAME__ = config.__REFVER__ + "_" + config.__GENENAME__
    # fetch data from 1000GP
    getData(filepath)

def main():
```

```
    print("*****STARTING SELECTION*****")
    config.__FOLDERPATH__ = os.getcwd()[:-(len('src/'))]
    errCode = selectGene(config.__FOLDERPATH__)


'''
FILE: fetch.py
PURPOSE: finds location of gene of interest; then, finds closest protein-encoding genes (NOT pseudogenes) to find range to search
INPUT: none
OUTPUT: error code (-1 if error)
CREATED BY: Anne-Sophie Fratzscher
NOTES: Implementation based on https://www.ncbi.nlm.nih.gov/dbvar/content/tools/entrez/
'''
import config
import pandas as pd
import sys
from Bio import Entrez
import xmltodict

'''
FUNCTION: setConfigValues(p)
PURPOSE: sets config values based on info found either in db or in file
INPUT: p (row of info from file or db)
OUTPUT: none
'''
def setConfigValues(p):
    config.__CHRVERSION__ = p['ChrAccVer']
    chrom = ((config.__CHRVERSION__[4:].lstrip("0")).split(".", 1))[0]
    config.__CHR__ = int(chrom)
    start = int(p['ChrStart']) + 1
    end = int(p['ChrStop']) + 1
    if start > end: # need to do this check because sometimes the values are flipped?
        temp = start
        start = end
        end = temp
    config.__GENESTART__ = int(start)
    config.__GENEEND__ = int(end)

'''
FUNCTION: eSearch(ext, nt)
PURPOSE: makes call to database
INPUT: ext (term to search) and nt (database to search)
OUTPUT: results from search
```

```
'''
def eSearch(ext, nt):
	Entrez.email = config.__EMAIL__
	paramEutils = {'usehistory': 'Y'} # use entrez search history to cache results

	if nt == 'nucleotide':
		db = 'nucleotide'
		term = ext
	else:
		db = 'gene'
		term = '("homo sapiens"[Organism]) AND ' + ext

	eSearch = Entrez.esearch(db=db, term=term, sort='Location', **paramEutils)

	res = Entrez.read(eSearch)

	paramEutils['WebEnv'] = res['WebEnv']
	paramEutils['query_key'] = res['QueryKey']
	paramEutils['rettype'] = 'xml' #report as xml
	paramEutils['retstart'] = 0

	result = Entrez.esummary(db=db, **paramEutils)
	xml = result.read()

	# convert xml to python dict object for convenient parsing
	dsdocs = xmltodict.parse(xml)
	return dsdocs

'''
FUNCTION: getGeneRange()
PURPOSE: finds start and end nucelotide for gene
INPUT: none
OUTPUT: error code (if cnnot be found)
'''
def getGeneRange():
	dsdocs = eSearch(config.__GENENAME__, "")

	fail = False
	errCode = 0

	try:
		dsdocs['eSummaryResult']['DocumentSummarySet']
	except KeyError: #search failed -> return that gene not found
		fail = True
```

```
                errCode = 1
                return(errCode)

        if not fail:
                #get set of dbVar DocumentSummary (dsdocs) and print report for each (ds)
                for ds in dsdocs
['eSummaryResult']['DocumentSummarySet']['DocumentSummary']:
                        if ds['Name'] == config.__GENENAME__:
                                for p in ds['LocationHist']['LocationHistType']:
                                        if p['ChrAccVer'][:7] == 'NC_0000':
                                                if (config.__REFVER__ == '37'): #want first 105
(since is sorted)
                                                        config.__ANNOTATIONRELEASE__ = '105'
                                                        if (p['AnnotationRelease'] == '105'):
                                                                setConfigValues(p)
                                                elif (config.__REFVER__ == '38'):
                                                        config.__ANNOTATIONRELEASE__ =
p['AnnotationRelease']

                                                        setConfigValues(p)
                                                        break

        return(errCode)

'''
FUNCTION: getLength()
PURPOSE: gets length of chromosome
INPUT: none
OUTPUT: length of chromosome
'''
def getLength():
        chromV = config.__CHRVERSION__
        chromosome = config.__CHR__

        if config.__REFVER__ == '38': # search nucleotide database
                dsdocs = eSearch(chromV, 'nucleotide')
                for ds in dsdocs['eSummaryResult']['DocSum']['Item']:
                        if (ds['@Name'] == 'Length'):
                                length = ds['#text']
                return(int(length))
        elif config.__REFVER__ == '37': # read from file
                versionFile = 'GRCh37_version_length.txt'
                data = pd.read_csv(versionFile, sep="\t")
                length = data.iloc[int(chromosome)-1]['length']
                return(int(length))
```

```
'''
FUNCTION: getClosest(upDown, length)
PURPOSE: gets length of chromosome
INPUT: upDown (look upstream of downstream of gene), length of chromosome
OUTPUT: none
'''
def getClosest(upDown, length): # looks for nearest protein-coding gene (NOT pseudogene)
	increment = 100000 # default is search 100,000 bases away

	check = 0
	while (check == 0): # if not found, search further
		if upDown == 'up':
			start = config.__GENESTART__ - increment
			end = config.__GENESTART__
		elif upDown == 'down':
			start = config.__GENEEND__
			end = config.__GENEEND__ + increment

		if (start < 1):
			config.__START__ = 1
			return
		elif (end > length):
			config.__END__ = int(length)
			return
		df = pd.DataFrame(columns=['name', 'start', 'end'])
		chromosome = config.__CHRVERSION__
		ext = (chromosome.split(".", 1)[0]
			+ "[nucl_accn] AND (" + str(start) + "[CHRPOS]"
			+ " : " + str(end) + "[CHRPOS]" + ') AND ("genetype protein
coding"[Properties])')
		dsdocs = eSearch(ext, "")

		fail = False
		try:
			dsdocs['eSummaryResult']['DocumentSummarySet']
		except KeyError: #search failed (nothing found)
			fail = True
			increment += 100000

		successful = False
		if not fail:
			try: # only 1 gene found (edge case)
```

```
                    name =
dsdocs['eSummaryResult']['DocumentSummarySet']['DocumentSummary']['Name']
                    successful = True
                except TypeError: # multiple genes found
                    for ds in dsdocs
['eSummaryResult']['DocumentSummarySet']['DocumentSummary']:
                        name = ds['Name']
                        for p in ds['LocationHist']['LocationHistType']:
                            if (p['ChrAccVer'][:2] == 'NC'):
                                if (p['AnnotationRelease'] ==
config.__ANNOTATIONRELEASE__):
                                    if (p['ChrAccVer'] ==
config.__CHRVERSION__):
                                        fStart = int(p['ChrStart']) + 1
                                        fEnd = int(p['ChrStop']) + 1
                                        if fStart > fEnd:
                                            temp = fStart
                                            fStart = fEnd
                                            fEnd = temp
                                        if (fStart > start):
                                            df =
df.append({'name': name, 'start': fStart, 'end' : fEnd}, ignore_index = True)
                if successful:
                    for p in dsdocs
['eSummaryResult']['DocumentSummarySet']['DocumentSummary']['LocationHist']['LocationHis
tType']:
                        if (p['ChrAccVer'][:2] == 'NC'):
                            if (p['AnnotationRelease'] ==
config.__ANNOTATIONRELEASE__):
                                if (p['ChrAccVer'] ==
config.__CHRVERSION__):
                                    fStart = int(p['ChrStart']) + 1
                                    fEnd = int(p['ChrStop']) + 1
                                    if fStart > fEnd: # sometimes end
and start swapped in db
                                            temp = fStart
                                            fStart = fEnd
                                            fEnd = temp
                                    if (fStart > start):
                                        df = df.append({'name':
name, 'start': fStart, 'end' : fEnd}, ignore_index = True)

                # remove gene from df if in list
                df = df[df.name != config.__GENENAME__]
```

```
				if (df.empty): # if empty, continue searching
					increment+=increment #doubles increment
					df = df.drop(df.index, inplace=True) # clear data frame at end
				else: # else, finish searching
					check = 1

		if upDown == 'up':
			df = df.sort_values('end', ascending=False)
			df = df.reset_index(drop = True)
			config.__START__ = int((df['end'][0]) + 1)
		elif upDown == 'down':
			df = df.sort_values('start')
			df = df.reset_index(drop = True)
			config.__END__ = (df['start'][0]) - 1
		df = df.drop(df.index, inplace=True) # clear data frame at end

def main():
		print("*****STARTING FETCHING*****")
		try:
			errCode = getGeneRange() #gets range of gene
		except TypeError:
			return(-1)
		if (errCode == 0):
			length = getLength()
			getClosest('up', length) # gets upstream location
			getClosest('down', length)
			return(0)
		else:
			return(-1)

'''
FILE: read_vcf.py
PURPOSE: reads vcf files
INPUT: path to file
OUTPUT: opened vcf file
CREATED BY: GitHub user dceoy
NOTES: repository can be found here:
https://gist.github.com/dceoy/99d976a2c01e7f0ba1c813778f9db744
'''
#!/usr/bin/env python

import io
import os
```

```
import pandas as pd

def read_vcf(path):
    with open(path, 'r') as f:
        lines = [l for l in f if not l.startswith('##')]
    return pd.read_csv(
        io.StringIO(''.join(lines)),
        dtype={'#CHROM': str, 'POS': int, 'ID': str, 'REF': str, 'ALT': str,
               'QUAL': str, 'FILTER': str, 'INFO': str},
        sep='\t'
    ).rename(columns={'#CHROM': 'CHROM'})


'''
FILE: cleaner.py
PURPOSE: removes SNPs that are out of specified range from file
                    and removes samples that have >1 heterozygous SNP
INPUT: none
OUTPUT: cleaned csv file named "cleaned_1000G_chr1_159203314-159283887.vcf"
CREATED BY: Anne-Sophie Fratzscher
'''
import config
import read_vcf
from pathlib import Path
import numpy as np
import pandas as pd

'''
FUNCTION: getIndividuals()
PURPOSE: gets ID for 2504 individuals from phase 3 of 1000GP
INPUT: none
OUTPUT: list of individuals from phase 3 of 1000GP
'''
def getIndividuals():
        # this file holds ID for the 2504 individuals
        indFile = 'integrated_call_samples_v3.20130502.ALL.panel.txt'
        data = pd.read_csv(indFile, sep="\t")
        indi = []
        for i in data['sample']:
                indi.append(i)
        return indi

def manualclean(df):
        df = df[df['ID'] != 'rs529460769']
```

```
	return df
'''
FUNCTION: clean()
PURPOSE: removes samples that are not part the 2504
		individuals for phase 3 AND removes samples
		with > 1 heterozygous SNP
INPUT: list of individuals in 1000GP phase 3
OUTPUT: cleaned csv file named "cleaned_1000G_chr1_159203314-159283887.vcf"
'''
def clean(individuals):
	df = read_vcf.read_vcf(filename)

	copy = rangeSelection(df)

	columns = copy.columns
	infoNames = {'CHROM','POS','ID','REF','ALT',
		'QUAL','FILTER','INFO','FORMAT'}

	# get all sample names
	initsamples = []
	for i in copy.columns:
		if not (i in infoNames):
			initsamples.append(i)

	cleanedsamples = []
	dups = []

	# get samples that are in indFile AND with at most 1 hetero SNP within range
	for i in initsamples:
		if i in individuals and i not in dups:
			# gets counts for different values (0|0, 0|1, 1|0, ...)
			count = copy[i].value_counts()
			totalcount = df[i].value_counts()

			# if homozygous (ie. 0|0, 1|1, 2|2, ... 6|6), dont count
			totalSNPS = 0
			rangeSNPS = 0

			# get range SNPS first
			for c in range(0, len(count)):
				if (count.index[c][0] != count.index[c][-1]):
						rangeSNPS += count[c]

			if rangeSNPS <= 1:
```

```
                    cleanedsamples.append(i)
                    # get total SNPS if part of clean sample
                    for c in range(0, len(totalcount)):
                        if (totalcount.index[c][0] != totalcount.index[c][-1]):
                                totalSNPS += totalcount[c]
                    df.at[-1,i] = totalSNPS
                dups.append(i)

    # remove samples from file that are not part of cleanedsamples list
    for i in initsamples:
        if not(i in cleanedsamples):
            del df[i]

    df = manualclean(df)
    df.to_csv(cleanedName, sep="\t", mode='a', index=False)
'''
FUNCTION: rangeSelection(df)
PURPOSE: removes SNPs that are outside of range
INPUT: df
OUTPUT: returns ranged df
'''
def rangeSelection(df):
    mask = (df['POS'] >= config.__GENESTART__) & (df['POS'] <= config.__GENEEND__)
    df = df.loc[mask]
    return df

def main():
    print('*****STARTING CLEANER*****')
    global filename
    global cleanedName
    filename = config.__FILEPATH__ + config.__FILENAME__
    cleanedName = config.__FILEPATH__ + "cleaned_" + config.__FILENAME__

    # if already cleaned
    fileCheck = Path(cleanedName)
    if fileCheck.is_file():
        return

    individuals = getIndividuals()
    clean(individuals)

'''
FILE: haplotype.py
PURPOSE: obtain haplotypes
```

```
INPUT: none
OUTPUT: csv file named "haplotypes_1000G_chr1_159203314-159283887.vcf"
CREATED BY: Anne-Sophie Fratzscher
'''
import config
import sequence
import pandas as pd
import numpy as np
import math
from pathlib import Path

'''
FUNCTION: getHaplotypes(filename)
PURPOSE: gets haplotypes
INPUT: filename
OUTPUT: haplotype csv
'''
def getHaplotypes(filename):
	df = pd.read_csv(filename, sep="\t")

	# only keep samples, SNP, and ref/alt nucleotide
	infoNames = {'CHROM', 'QUAL','FILTER','INFO','FORMAT'}
	for i in infoNames:
		del df[i]

	# call sequence file to get FASTA sequence and add
	df = sequence.main(df)
	df = df.dropna(axis=0, how="all").reset_index(drop=True) # delete rows with all nan

	# get unambiguous haplotypes
	df = getUnambiguous(df)

	# split into maternal and paternal haplotype
	df = splitHaplotypes(df)

	# replace with N all outside of start/end
	for i in df.columns:
		if i not in ['POS', 'ID', 'REF', 'ALT']:
			startval = int(df.loc[len(df.index)-3][i])
			endval = int(df.loc[len(df.index)-2][i])
			if startval > config.__START__:
				startidx = df.index[df['POS'] == startval-1].tolist()
				if startidx:
					idx = startidx[0]
```

```
                        df.loc[0:idx, i] = 'N'
                if endval < config.__END__:
                    endidx = df.index[df['POS'] == endval+1].tolist()
                    if endidx:
                        idx = endidx[0]
                        df.loc[idx:len(df.index) - 4, i] = 'N'

        # replace values with nucleotides (only if SNP -> if ref, keep as '-')
        # also count indel
        for (index,row) in df.iterrows():
            if not (index > len(df.index) - 5): # ignore last 5 rows (start, end, #SNPS...)
                if not df.loc[index]['ID'] is None:
                    ref = ['-']
                    reflen = len(row['REF'])
                    alt = row['ALT'].split(',')
                    # list of nucleotides (pos 0 = ref, pos 1 - 6 = alts)
                    nt = ref + alt
                    for i in range(4,len(row)):
                        if not df.iloc[index, i] == 'N':
                            start = df.iat[-3, i]
                            end = df.iat[-2, i]
                            indel = df.iat[-1, i]
                            # if last SNP is ALT and is full length haplotype,
change end site to position of LAST SNP
                            if (index == len(df.index)-6): # if last row
                                if (end == config.__END__): # if is full length
haplotype
                                    if not (df.iat[index, i] == '0|0'): # if
last SNP is alt, alter end site
                                        val = row[i]
                                        if "p_" in row.index[i]:
                                            if (val[0] != '0'):
                                                df.iat[-2, i] =
int(df.loc[index]['POS']) # if alt, set POS = last SNP pos
                                                df.iat[index+1,
i] = "N"
                                        else:
                                            if (val[-1] != '0'):
                                                df.iat[-2, i] =
int(df.loc[index]['POS']) # if alt, set POS = last SNP pos
                                                df.iat[index+1,
i] = "N"
```

```
						if not (df.iat[index, 0] < start) or (df.iat[index, 0] >
end):
							val = row[i]
							if (df.iat[index,i] == '.|.' or df.iat[index,i] ==
'.'): # if cannot make call for locus, set to 'Q'
								df.iat[index, i] = 'Q'
							else:
								if "p_" in row.index[i]: # first
occurence
									df.iat[index,i] = nt[int(val[0])]
									if (nt[int(val[0])] != '-'):
										df.iat[-1, i] +=
(len(nt[int(val[0])]) - reflen)
								else:
									df.iat[index,i] = nt[int(val[-
1])]

									if (nt[int(val[-1])] != '-'):
										df.iat[-1, i] +=
(len(nt[int(val[-1])]) - reflen)
	# sort by length
	df = df.rename(columns = {'POS': 'idx'})
	df = df.set_index('idx')
	df = sortHaplotypes(df)

	df.to_csv(haplotypeFile, sep="\t", mode='a', index = False)

'''
FUNCTION: getUnambiguous(df)
PURPOSE: gets part of haplotype that is unambiguous
INPUT: dataframe
OUTPUT: dataframe of unambiguous haplotypes with start and end row
'''
def getUnambiguous(df):
	homozy = ["0|0", "1|1", "2|2", "3|3", "4|4", "5|5", "6|6"]

	SNPS = df.tail(1)

	# add rows to store start and end for haplotype
	df.loc[len(df)] = "-" # start
	df.loc[len(df)] = "-" # end

	d = df.dropna()
	d = d.mask(d.isin(homozy)).drop(d.tail(3).index)
	d = d.reset_index(drop=True)
```

```
        gene = d[(d['POS'] > config.__GENESTART__) & (d['POS'] <= config.__GENEEND__)] #
SNPS within gene
        gene = gene.reset_index(drop=True)
        before = d.drop(d[d['POS'] > config.__GENESTART__].index) # SNPs before gene
        after = d.drop(d[d['POS'] < config.__GENEEND__].index) # SNPs after gene
        after = after.reset_index(drop=True)
        start = before.notna()[::-1].idxmax()
        end = after.notna().idxmax()
        endsecond = after.notna().cumsum().eq(2).idxmax() #index of second SNP after gene
        within = gene.notna().sum(axis = 0) # number of hetero SNP in gene (0 or 1)

        for i in df.columns:
                if i in ['POS', 'ID' 'REF', 'ALT']:
                        start[i] = "-"
                        end[i] = "-"
                else:
                        if within[i] == 1: # if 1 hetero SNP in gene (dont add another hetero)
                                # get start site
                                if(pd.isna(before.iloc[start[i]][i])): # if no SNP before, set to start
                                        df.iloc[-2][i] = config.__START__
                                else:
                                        if (start[i]+1 < len(before)):
                                                df.iloc[-2][i] = (before.iloc[start[i]]['POS']) + 1
                                        else:
                                                df.iloc[-2][i] = config.__START__
                                # get end site
                                if(pd.isna(after.iloc[end[i]][i])):
                                        df.iloc[-1][i] = config.__END__
                                else:
                                        df.iloc[-1][i] = (after.iloc[end[i]]['POS']) - 1
                        else:
                                # get start site
                                if(pd.isna(before.iloc[start[i]][i])): # if no SNP before, set to start
                                        df.iloc[-2][i] = config.__START__
                                else:
                                        if (start[i]+1 < len(before)):
                                                df.iloc[-2][i] = (before.iloc[start[i]]['POS']) + 1
                                        else:
                                                df.iloc[-2][i] = config.__START__
                                # get end site -> includes 1 hetero SNP
                                if(pd.isna(after.iloc[endsecond[i]][i])):
                                        df.iloc[-1][i] = config.__END__
                                else:
```

```
                                        df.iloc[-1][i] = (after.iloc[endsecond[i]]['POS']) - 1

        df.iloc[-1]["POS"] = 'end'
        df.iloc[-2]["POS"] = 'start'
        df.iloc[-3]["POS"] = 'numHeteroSNPs'
        df.iloc[-3]["ID"] = 0
        df.iloc[-3]["REF"] = 0
        df.iloc[-3]["ALT"] = 0
        df.iloc[-1]["ID"] = 0
        df.iloc[-1]["REF"] = 0
        df.iloc[-1]["ALT"] = 0
        df.iloc[-2]["REF"] = 0
        df.iloc[-2]["ALT"] = 0
        df.iloc[-2]["ID"] = 0

        # save added/removed nucleotide length
        df.loc[len(df)] = 0 # indel length
        df.iloc[-1]["POS"] = 'indel_num'

        return df

'''
FUNCTION: splitHaplotypes(df)
PURPOSE: splits into maternal and paternal haplotypes
INPUT: dataframe
OUTPUT: dataframe with maternal and paternal haplotypes
'''
def splitHaplotypes(df):
        for i in df.columns:
                if not (i == 'POS' or i == 'ID' or i == 'REF' or i == 'ALT'):
                        df['m_'+i] = df[i]
                        df.rename(columns={i: 'p_'+i}, inplace=True)
        return df

'''
FUNCTION: sortHaplotypes(df)
PURPOSE: sorts haplotypes by length
INPUT: dataframe
OUTPUT: sorted dataframe by haplotype length
'''
def sortHaplotypes(df):
        df = df.transpose()
        df['length'] = (df['end'] - df['start']) + 1
        excluded = df.head(3)
```

```
        included = df.iloc[3:]
        included = included.sort_values(by ='length', ascending=[False]) #longest at top
        df = pd.concat([excluded, included])
        df = df.reset_index()
        df = df.rename(columns = {'index': 'POS'})

        return df

def main():
        print('*****STARTING HAPLOTYPES*****')
        global haplotypeFile
        filename = config.__FILEPATH__ + "cleaned_" + config.__FILENAME__
        haplotypeFile = config.__FILEPATH__ + "haplotypes_" + config.__FILENAME__

        # if already have haplotypes
        fileCheck = Path(haplotypeFile)
        if fileCheck.is_file():
                return

        getHaplotypes(filename)

'''
FILE: sequence.py
PURPOSE: get sequence around SNP
INPUT: df
OUTPUT: df with sequence added
CREATED BY: Anne-Sophie Fratzscher
'''

import sys
from Bio import Entrez, SeqIO
import xmltodict
import config
import pandas as pd
from pathlib import Path
from toolz import interleave

def eFetch():
        Entrez.email = config.__EMAIL__
        term = str(config.__CHRVERSION__)

        handle = Entrez.efetch(db="nucleotide", id=term,
                seq_start=config.__START__, seq_stop=config.__END__,
                strand=1, rettype="gb",retmode="text")
```

```
        record = SeqIO.read(handle, "genbank")
        handle.close()
        return record

def getFASTA():
        record = eFetch()
        seq = str(record.seq)
        return seq

def replace(df, seq):
        pos = []
        location = []
        other = ['POS', 'ID', 'REF', 'ALT']
        for (index, row) in df.iterrows():
                if index < len(df.index)-1:
                        pos.append(int(float(df.iloc[index]['POS'])) - config.__START__)
        pos.append(config.__END__ - config.__START__)

        sequence = []
        for i in range(0, len(pos)):
                if i == 0:
                        seqtoadd = seq[0:pos[i]]
                        loc = config.__START__
                elif i == (len(pos)-1):
                        seqtoadd = seq[(pos[i-1]+1):(pos[i]+1)]
                        loc = config.__START__ + pos[i-1]+1
                else:
                        seqtoadd = seq[(pos[i-1]+1):pos[i]]
                        loc = config.__START__ + pos[i-1]+1
                if seqtoadd == "":
                        sequence.append(None)
                        location.append(None)
                else:
                        sequence.append(seqtoadd)
                        location.append(loc)

        rowseq = []
        for i in range(0, len(sequence)):
                rowseq.append([sequence[i]] * (len(df.columns)-2))

        new = pd.DataFrame(rowseq)
        new.insert(0, 'POS', location)
        new.insert(1, 'ID', None)
```

```
	new.columns = df.columns
	df = pd.DataFrame(interleave([new.values, df.values]))
	df.columns = new.columns
	return df

def main(df):
	seq = getFASTA()
	df = replace(df, seq)
	return df

'''
FILE: distinct.py
PURPOSE: obtain distinct haplotypes
INPUT: none
OUTPUT: 2 csv files -> 1 with counts, 1 with distinct haplotypes
CREATED BY: Anne-Sophie Fratzscher
'''
import config
from pathlib import Path
import pandas as pd
import numpy as np
import math
import itertools

'''
FUNCTION: getCounts(filename)
PURPOSE: gets count for each haplotype (number of times either as duplicate or
	as subcomponent)
INPUT: filename
OUTPUT: csv with counts, sorted by number of counts (descending)
'''
def getCounts(filename):
	# NOTE: longest haplotype first
	df = pd.read_csv(filename, sep="\t")
	info = df.head(3) #stores id, ref, alt (not used but important)
	df = df.drop(df.head(3).index)
	start = df['start']
	end = df['end']
	indel_num = df['indel_num']
	del df['start']
	del df['end']
	del df['indel_num']
	lengthdf = df['length'] # append later
	posdf = df['POS']
```

```
		del df['POS']
		del df['length']
		del df['numHeteroSNPs']

		d = df.loc[:, ~df.columns.str.contains('^Unnamed')] # remove conserved parts

		# get list of samples and list of haplotypes
		x = d.to_string(header=False, index=False,
										index_names=False).split('\n')
		haplotypes = [''.join(ele.split()) for ele in x]

		y = posdf.to_string(header=False, index=False).split('\n')
		samples = [''.join(ele.split()) for ele in y]

		identical = samples.copy()
		subSample = samples.copy()
		counts = [0] * len(identical) # DOESNT include sample
		longest = [0] * len(identical)
			# for a (longer haplotype being compared), 0 intially, 1 if already found longest
subsequence,
														# 2 if
no longest subsequence found 0 after comparing to all other haplotypes
		found = [0] * len(identical)
			# for b (shorter haplotype being compared), 0 initially, 1 if subsamples already
found for haplo

		for ((aidx,a),(bidx,b)) in itertools.combinations(enumerate(haplotypes), 2):
			if not (haplotypes[aidx] is None or haplotypes[bidx] is None):
				if a == b: # identical haplotypes
					haplotypes[bidx] = None
					identical[aidx] = identical[aidx] + ", " + identical[bidx]
					identical[bidx] = None
					subSample[aidx] = subSample[aidx] + ", " + subSample[bidx]
				else:
					if ((b == (len(samples) - 1)) and (longest[aidx] == 0)): # if didnt find
subseqeuence, set longest to 2
						longest[aidx] = 2
					if (longest[aidx] != 2 and found[aidx] != 1):
					# compare SNP by SNP
						occ = [b.find('A'), b.find('G'), b.find('C'),
										b.find('T'), b.find('-')] #,
len(b)+1] # find first non N
						startidx = min(i for i in occ if i >= 0) # if one of them is 0,
means no N
```

```
                                        endidx = max(b.rfind('A'), b.rfind('G'), b.rfind('C'),
                                                        b.rfind('T'), b.rfind('-'))
#len(b)-1)
                                        if b[startidx:endidx+1] == a[startidx:endidx+1]:
                                            if (longest[aidx] == 0):
                                                if subSample[aidx] == None:
                                                    subSample[bidx] = samples[aidx]
                                                elif subSample[bidx] == None:
                                                    subSample[bidx] = subSample[aidx]
                                                else:
                                                    subSampleList =
set(list(subSample[bidx].split(", "))).union(set(list(subSample[aidx].split(", "))))
                                                    for i in identical[aidx].split(", "):
                                                        subSampleList.remove(i)
                                                    subSample[bidx] = ',
'.join(subSampleList)

                                                    subSample[aidx] = None
                                                longest[aidx] = 1
                                                found[bidx] = 1

    # get counts
    for i in range(0, len(counts)):
        if subSample[i] is None:
            subSample[i] = identical[i]
        if subSample[i] == samples[i] and (identical[i] is not None):
            subSample[i] = identical[i]
        counts[i] = len(list(subSample[i].split(", ")))

    df.insert(loc=0, column='sampleID', value = samples)
    df.insert(loc=1, column='subsamples', value=subSample)
    df.insert(loc=2, column='identical', value = identical)
    df['start'] = start
    df['end'] = end
    df['length'] = lengthdf + indel_num
    df['counts'] = counts

    # format info
    info.rename(columns={'POS':'sampleID'}, inplace=True)
    del info['indel_num']
    info.insert(loc=1, column='haplotypeID', value = '-')
    info.insert(loc=2, column='subsamples', value = '-')
    info.insert(loc=3, column='identical', value = '-')
    info['counts'] = 0
    del info['numHeteroSNPs']
```

```
	# remove identical haplotypes
	df = df.dropna(axis=0, subset=['identical'])
	df.insert(loc=1, column='haplotypeID', value=["HAP" + str(i+1) for i in
range(len(df.index))])
	# sort by count number (descending)
	countsorted = df.sort_values(by='counts', ascending=False)
	df2 = pd.concat([info, countsorted])
	df2.to_csv(countFile, sep="\t", mode='a', index = False)

	getDistinct(df, info)

'''
FUNCTION: getDistinct(df, samples)
PURPOSE: creates csv with only distinct haplotypes, with samples
	 with that haplotype stored
INPUT: df, info (from count method)
OUTPUT: csv of distinct haplotypes, sorted by length (descending)
'''
def getDistinct(df, info):
	#sort by length
	df = df.sort_values(by='length', ascending=False)
	df = pd.concat([info, df])

	df.to_csv(distinctFile, sep="\t", mode='a', index = False)

def main():
	print('*****STARTING DISTINCT*****')
	global countFile
	global distinctFile
	filename = config.__FILEPATH__ + "haplotypes_" + config.__FILENAME__
	countFile = config.__FILEPATH__ + "count_" + config.__FILENAME__
	distinctFile = config.__FILEPATH__ +  "distinct_" + config.__FILENAME__

	# if already have counts
	fileCheck = Path(countFile)
	if fileCheck.is_file():
		return

	getCounts(filename)

'''
FILE: popcounts.py
PURPOSE: obtain populations from distinct haplotypes
```

INPUT: none
OUTPUT: 2 csv files with population counts called
    1) mostfreq_chr1_159203314-159283887.vcf
    2) identical_chr1_159203314-159283887.vcf
CREATED BY: Anne-Sophie Fratzscher
'''
import config
import pandas as pd
import numpy as np
import math
from pathlib import Path
import time

'''
FUNCTION: getPopsMostFreq(filename)
PURPOSE: gets population/ superpopulation count based on
    subsamples found (used for most frequent)
INPUT: filename
OUTPUT: csv called "mostfreq_chr1_159203314-159283887.vcf"
'''
def getPopsMostFreq(filename):
    popFile = config.__FILEPATH__ + config.__FOLDERNAME__ + "/mostfreq_" +
config.__FILENAME__
    # if already have populations
    fileCheck = Path(popFile)
    if fileCheck.is_file():
        return

    df = pd.read_csv(filename, sep="\t")
    df = replacePop(df, 'subsamples')
    df = df.reset_index(drop=True)
    info = df.head(3) #stores id, ref, alt (not used but important)
    df = df.drop(df.head(3).index)

    df = df.sort_values(by='counts', ascending=False)
    df = pd.concat([info, df])
    df.to_csv(popFile, sep="\t", mode='a', index = False)

'''
FUNCTION: getPopsIdentical(filename)
PURPOSE: gets population/ superpopulation count based on
    identical samples found (used for multiple populations)
INPUT: filename

```
OUTPUT: csv called "identical_chr1_159203314-159283887.vcf"
'''
def getPopsIdentical(filename):
	popFile = config.__FILEPATH__ + config.__FOLDERNAME__ + "/identical_" +
config.__FILENAME__

	# if already have populations
	fileCheck = Path(popFile)
	if fileCheck.is_file():
		return

	df = pd.read_csv(filename, sep="\t")
	df = replacePop(df, 'identical')
	df.to_csv(popFile, sep="\t", mode='a', index = False)

'''
FUNCTION: replacePop(df, replaceType)
PURPOSE: algortihm to count population/superpopulation
INPUT: dataframe and replaceType (identical or subsample)
OUTPUT: dataframe
'''
def replacePop(df, replaceType):
	tf = pd.read_csv('integrated_call_samples_v3.20130502.ALL.panel.txt', sep="\t")

	# add columns for these pops
	for x in config.__POPS__:
		df[x] = 0
	df['numberOfPops'] = 0

	# add columns for super pops
	for x in config.__SUPERPOPS__:
		df[x] = 0
	df['numberOfSuperpops'] = 0

	# remove ID, ALT, REF
	info = df.head(3) #stores id, ref, alt (not used but important)
	df = df.drop(df.head(3).index)
	df = df.reset_index(drop=True)

	# get pops (using info from 2504 gp file)
	xidx = 0
	for x in df[replaceType]:
		dup = [] # to account for 2 copies of DNA
		dup_pop = []
```

```
			dup_super_pop = []
			if not (pd.isnull(x)):
				x = x.split(", ")
				for i in range(0, len(x)):
					# look for sample in "samples" df and get pop, super_pop, gender
					if x[i][2:] not in dup:
						idx = tf[tf['sample'] == x[i][2:]].index.tolist()
						sample_pop = tf.iloc[idx[0]]['pop']
						sample_super_pop = tf.iloc[idx[0]]['super_pop']

						# increment count if new pop
						if sample_pop not in dup_pop:
							df.at[xidx, 'numberOfPops']+=1
						# increment count if new super pop
						if sample_super_pop not in dup_super_pop:
							df.at[xidx, 'numberOfSuperpops']+=1

						df.at[xidx, sample_pop] += 1 #increment pop
						df.at[xidx, sample_super_pop] +=1 # increment superpop

						#append gender, pop, superpop
						dup_pop.append(sample_pop)
						dup_super_pop.append(sample_super_pop)

					dup.append(x[i][2:])
			xidx+=1
		df = pd.concat([info, df])
		return df

def main():
	print("*****STARTING POP COUNTER*****")
	distinctFile = config.__FILEPATH__ + config.__FOLDERNAME__ + "/distinct_" +
config.__FILENAME__

	getPopsMostFreq(distinctFile)
	getPopsIdentical(distinctFile)

'''
FILE: visualization.py
PURPOSE: visualizes
INPUT: none
OUTPUT: visualization as pie graph
CREATED BY: Anne-Sophie Fratzscher
'''
```

```
import config
import pandas as pd
import numpy as np
import math
from pathlib import Path
import time
import matplotlib.pyplot as plt
import os

def getVisualization(df, name, cscopy, fullFlag):
	if df.empty: # if nothing found
		plt.title("No %s".center(80) %(name))
		if fullFlag == 0: # NOT full length haplotype
				graphname = name + ".png"
		else:
			graphname = "full_" + name + ".png"
		plt.savefig(visualizationFolder + graphname)
		plt.clf()

	else:
		graphnum = 0

		#graph coloring scheme
		cmap = plt.get_cmap("magma")

		for (index, x) in df.iterrows():
			graphnum += 1
			pops = []
			values = []
			labeling = []
			cs = []
			for i in range(0, len(x)):
				if i == (len(x) - 4): # if length
					length = str(int(x[i]))
				elif i == (len(x) - 3): # if start
					start = str(int(x[i]))
				elif i == (len(x) - 2): # if end
					end = str(int(x[i]))
				elif i == (len(x) - 1): # if haplotypeID
					haploID = str(x[i])
				elif x[i] != 0:
					values.append(int(x[i]))
					pops.append(df.columns[i])
					labeling.append(str(df.columns[i]) + "," + str(int(x[i])))
```

```
                                        cs.append(cscopy[i])

                        csfull = cmap(cs)

                        # visualize (save as file)
                        data = values

                        fig, ax = plt.subplots()
                        ax.axis('equal')
                        pie = ax.pie(data, radius = 1, labeldistance=1.005, labels=labeling,
                rotatelabels =True, startangle=90,counterclock=False,
                colors = csfull, textprops={'fontsize': 8})

                        graphType = ''
                        if (fullFlag != 0): # 0 if not full, not 0 if full
                                graphType = 'Full length'
                        if name == 'most_frequent':
                                graphType = 'Most frequent'
                        else:
                                graphType += ' multiple ' + name

                        plt.title("%s haplotype (ID: %s): \nlength %s (chr %s: nt %s
- %s)" %(graphType, haploID, length, config.__CHR__, start, end))
                        if fullFlag == 0: # NOT full length haplotype
                                graphname = name + str(graphnum) + ".png"
                        else:
                                graphname = "full_" + name + str(graphnum) + ".png"
                        plt.savefig(visualizationFolder + graphname, dpi=300)
                        plt.clf()

def visualizeFull():
        df = pd.read_csv(fullPopFile, sep="\t")
        lastSNPpos = df.columns[df.columns.get_loc("start")-2]
        info = df.head(3) #stores id, ref, alt (not used but important)
        df = df.drop(df.head(3).index)
        df = df[df['start'] <= config.__START__]
        df = df[df['end'] >= int(float(lastSNPpos))]
        df = pd.concat([info, df])
        df.to_csv(fullFile, sep="\t", mode='a', index = False)
        return(df)

def visualizeMultiplePopOnly(df):
        df = df.drop(df.head(3).index) # remove ID, REF, ALT
        df = df[df['numberOfPops'] > 1]
```

```
    return df

# drop rows (so only have number of rows = num)
def visualizeNumber(df, num):
    if not (num == 'all'):
        df = df.drop(df.index[num:len(df)])
    return df

def visualizePop(df, num, fullFlag):
    # get only columns for pop
    cols = [c for c in df.columns if c in config.__POPS__]
    cols.append('length')
    cols.append('start')
    cols.append('end')
    cols.append('haplotypeID')
    df = df[cols]
    df = visualizeNumber(df, num)
    getVisualization(df, "pop", config.__CSPOP__, fullFlag)
    return

def visualizeSuper(df, num, fullFlag):
    cols = [c for c in df.columns if c in config.__SUPERPOPS__]
    cols.append('length')
    cols.append('start')
    cols.append('end')
    cols.append('haplotypeID')
    df = df[cols]
    df = visualizeNumber(df, num)
    getVisualization(df, "super", config.__CSSUPER__, fullFlag)
    return

def visualizeMostFrequent(df):
    df = df.drop(df.head(3).index) # remove ID, REF, ALT
    df = df.sort_values(['counts', 'length'], ascending=[False, False])
        # picks longest most frequent haplotype
    df = df.drop(df.index[1:len(df)])
    cols = [c for c in df.columns if c in config.__POPS__]
    cols.append('length')
    cols.append('start')
    cols.append('end')
    cols.append('haplotypeID')
    df = df[cols]
    fullFlag = 0
    getVisualization(df, "most_frequent", config.__CSPOP__, fullFlag)
```

```
	return

def getInput(inputI):
	vistype = inputI

	if vistype == 'most frequent':
		full = 'n'
	else:
		full = 'y'

	if full == 'y' or full == 'yes':
		fileCheck = Path(fullFile)
		if fileCheck.is_file():
			df = pd.read_csv(fullFile, sep="\t")
		else:
			df = visualizeFull()
	elif full == 'n' or full == 'no':
		df = pd.read_csv(mostFreqFile, sep="\t")

	if vistype == 'most frequent':
		visualizeMostFrequent(df)
		return

	df = visualizeMultiplePopOnly(df) # only if in multiple populations
	num = 'all'

	fullFlag = 1
	if vistype == 'pop':
		visualizePop(df, num, fullFlag)
	elif vistype == 'super':
		visualizeSuper(df, num, fullFlag)
	# elif vistype == 'gender':
	#	visualizeGender(df, num, fullFlag)

def main():
	print("*****STARTING VISUALIZATION*****")
	global fullFile
	global fullPopFile
	global mostFreqFile
	global visualizationFolder
	mostFreqFile = config.__FILEPATH__ + config.__FOLDERNAME__ + "/mostfreq_" +
config.__FILENAME__
	fullPopFile = config.__FILEPATH__ + config.__FOLDERNAME__ + "/identical_" +
config.__FILENAME__
```

```
	fullFile = config.__FILEPATH__ + config.__FOLDERNAME__ + "/full_length_haplotypes_"
+ config.__FILENAME__

	visualizationFolder = (config.__FOLDERPATH__ + "/results/"
		+ config.__GENENAME__ + "/" + config.__FOLDERNAME__ + "/visualization/")
	if not (os.path.isdir(visualizationFolder)):
		try:
		   Path(visualizationFolder).mkdir(parents=True, exist_ok=True)
		except OSError:
		   print ("Creation of the directory %s failed" % visualizationFolder)

	# call getInput using grouping and mostfreq
	if config.__MOSTFREQ__ == 1:
		getInput('most frequent')
	for i in config.__GROUPING__:
		getInput(i)

'''
FILE: userfile.py
PURPOSE: creates clean output for user
INPUT: most frequent file
OUTPUT: sequence and meta files
CREATED BY: Anne-Sophie Fratzscher
'''
import config
import pandas as pd
import numpy as np
import math
from pathlib import Path
import time
import matplotlib.pyplot as plt
import os

def getMeta(df, metaFile):
	idx = df.columns.get_loc('start')
	meta = df.iloc[3:, idx:]
	haploID = df['haplotypeID']
	meta.insert(0, 'haplotypeID', haploID)
	meta.to_csv(metaFile, sep="\t", mode='a', index = False)

def getSequence(df, seqFile):
	idx = df.columns.get_loc(config.__POPS__[0])
	df = df.iloc[:, 0:idx]
	del df['subsamples']
```

```
	del df['identical']
	del df['sampleID']

	list = df.columns.tolist()
	list.insert(1, list.pop(list.index('length')))
	list.insert(2, list.pop(list.index('start')))
	list.insert(3, list.pop(list.index('end')))
	list.insert(4, list.pop(list.index('counts')))
	df = df.reindex(columns= list)

	# set length, start, end for REF, ALT (clear for ID)
	df.iloc[0, df.columns.get_loc("haplotypeID")] = 'SNV_ID'
	df.iloc[1, df.columns.get_loc("haplotypeID")] = 'REF'
	df.iloc[2, df.columns.get_loc("haplotypeID")] = 'ALT'
	df.iloc[0, df.columns.get_loc("length")] = '-'
	df.iloc[1, df.columns.get_loc("length")] = config.__END__ - config.__START__ + 1
	df.iloc[2, df.columns.get_loc("length")] = config.__END__ - config.__START__ + 1
	df.iloc[0, df.columns.get_loc("start")] = '-'
	df.iloc[1, df.columns.get_loc("start")] = config.__START__
	df.iloc[2, df.columns.get_loc("start")] = config.__START__
	df.iloc[0, df.columns.get_loc("end")] = '-'
	df.iloc[1, df.columns.get_loc("end")] = config.__END__
	df.iloc[2, df.columns.get_loc("end")] = config.__END__
	df.iloc[0, df.columns.get_loc("counts")] = '-'

	unnamed = [col for col in df.columns if 'Unnamed' in col]
	newnames = [None] * len(unnamed)
	df.rename(columns=dict(zip(unnamed, newnames)),inplace=True)

	haploID = df['haplotypeID']
	del df['haplotypeID']
	df = df.replace('N', ' ', regex=True)
	df.insert(0, "haploID", haploID)

	for i in df.columns:
		if i not in ['haplotypeID', 'length', 'start', 'end', 'counts', None, "", 'sampleID']:
			ref = df.loc[1][i]
			df[i] = df[i].replace('-', ref, regex=True) # replace - with nucleotide

	df.to_csv(seqFile, sep="\t", mode='a', index = False)

def outputFile(filename):
	seqFile = config.__FILEPATH__ + config.__FOLDERNAME__ + "/sequence_" +
config.__FILENAME__
```

```
	seqFile = seqFile[:-3] + 'vcf'
	metaFile = config.__FILEPATH__ + config.__FOLDERNAME__ + "/meta_" +
config.__FILENAME__
	metaFile = metaFile[:-3] + 'vcf'

	# if already have output
	fileCheck = Path(seqFile)
	if fileCheck.is_file():
		return

	df = pd.read_csv(filename, sep="\t")

	getMeta(df, metaFile)
	getSequence(df, seqFile)

def main():
	print('*****STARTING OUTPUT FORMATTING*****')
	countFile = config.__FILEPATH__ + config.__FOLDERNAME__ + "mostfreq_" +
config.__FILENAME__
	outputFile(countFile)
```
