# Supplementary material for: Cataloguing experimentally confirmed 80.7 kb-long ACKR1 haplotypes from the 1000 Genomes Project database
Source: BMC Bioinformatics. 2021 May 26;22:273. doi: 10.1186/s12859-021-04169-6 (PMC8150616; doi:10.1186/s12859-021-04169-6)
Supplement: Supplementary file 2 — Additional file 2 Table S1. Populations in the 1000GP database. [file 12859_2021_4169_MOESM2_ESM.pdf]

Cataloguing experimentally confirmed 80.7 kb-long *ACKR1* haplotypes from the 1000 Genomes Project database

Kshitij Srivastava, Anne-Sophie Fratzscher, Bo Lan and Willy Albert Flegel

Table S1. Populations in the 1000GP database*

Population Description	Population code	Super-population code	Individuals		
			Total	Analyzed	Percentage
Han Chinese in Beijing, China	CHB	EAS	103	83	80.58%
Japanese in Tokyo, Japan	JPT	EAS	104	78	75.00%
Southern Han Chinese	CHS	EAS	105	86	81.90%
Chinese Dai in Xishuangbanna, China	CDX	EAS	93	71	76.34%
Kinh in Ho Chi Minh City, Vietnam	KHV	EAS	99	71	71.72%
Utah Residents with Northern and Western European Ancestry	CEU	EUR	99	25	25.25%
Toscani in Italia	TSI	EUR	107	24	22.43%
Finnish in Finland	FIN	EUR	99	30	30.30%
British in England and Scotland	GBR	EUR	91	15	16.48%
Iberian Population in Spain	IBS	EUR	107	28	26.17%
Yoruba in Ibadan, Nigeria	YRI	AFR	108	88	81.48%
Luhya in Webuye, Kenya	LWK	AFR	99	77	77.78%
Gambian in Western Divisions in the Gambia	GWD	AFR	113	96	84.96%
Mende in Sierra Leone	MSL	AFR	85	66	77.65%
Esan in Nigeria	ESN	AFR	99	83	83.84%
Americans of African Ancestry in SW USA	ASW	AFR	61	32	52.46%
African Caribbeans in Barbados	ACB	AFR	96	58	60.42%
Mexican Ancestry from Los Angeles USA	MXL	AMR	64	25	39.06%
Puerto Ricans from Puerto Rico	PUR	AMR	104	21	20.19%
Colombians from Medellin, Colombia	CLM	AMR	94	33	35.11%
Peruvians from Lima, Peru	PEL	AMR	85	31	36.47%
Gujarati Indian from Houston, Texas	GIH	SAS	103	37	35.92%
Punjabi from Lahore, Pakistan	PJL	SAS	96	39	40.63%
Bengali from Bangladesh	BEB	SAS	86	44	51.16%
Sri Lankan Tamil from the UK	STU	SAS	102	49	48.04%
Indian Telugu from the UK	ITU	SAS	102	46	45.10%
		Total	2504	1336	53.35%

* Table adapted from the 1000 Genomes Project.[22]
