# Supplementary material for: Cataloguing experimentally confirmed 80.7 kb-long ACKR1 haplotypes from the 1000 Genomes Project database
Source: BMC Bioinformatics. 2021 May 26;22:273. doi: 10.1186/s12859-021-04169-6 (PMC8150616; doi:10.1186/s12859-021-04169-6)
Supplement: Supplementary file 5 — Additional file 5Table S4. Exonic SNV distribution in the 902 experimentally confirmed ACKR1 haplotypes. [file 12859_2021_4169_MOESM5_ESM.pdf]

Table S4. Exonic SNV distribution in the 902 experimentally confirmed *ACKR1* haplotypes

	SNVs in the *ACKR1* gene*																
ISBT allele	-67T>C (rs2814778)	125G>A (rs12075)	199C>T† (rs118062001)	265C>T (rs34599082)	298G>A† (rs13962)	366C>A‡ (rs563566546)	395G>A‡ (rs530992295)	455C>T‡ (rs147620003)	509C>T‡ (rs200287093)	655G>A‡ (rs146219376)	763C>T‡ (rs569108988)	832G>A‡ (rs200531663)	877G>A‡ (rs199599421)	892G>T‡ (rs201549518)	977C>T (rs17851570)	Haplotypes observed (n)‡	Haplotype length range (nucleotides)
*FY*01*	T	G	C	C	G	C	G	C	C	G	C	G	G	G	C	373	2241 - 80576
*FY*01*	T	G	T	C	G	C	G	C	C	G	C	G	G	G	C	13	3194 - 80572
*FY*01*	T	G	C	C	G	C	G	C	T	G	C	G	G	G	C	2	4869 - 9919
*FY*01*	T	G	C	C	G	C	G	C	C	G	T	G	G	G	C	1	14785
															Sub-total	389	
*FY*02*	T	A	C	C	G	C	G	C	C	G	C	G	G	G	C	113	1901 - 80576
*FY*02*	T	A	C	C	A	C	G	C	C	G	C	G	G	G	C	52	1901 - 60563
*FY*02*	T	A	C	C	A	C	G	T	C	G	C	G	G	G	C	1	35073
															Sub-total	166	
*FY*02N.01*	C	A	C	C	G	C	G	C	C	G	C	G	G	G	C	340	1977 - 80584
*FY*02N.01*	C	A	C	C	G	C	G	C	C	G	C	A	G	G	C	2	4643 - 15125
*FY*02N.01*	C	A	C	C	G	A	G	C	C	G	C	G	G	G	C	1	9918
*FY*02N.01*	C	A	C	C	G	C	G	C	C	A	C	G	G	G	C	1	4643
															Sub-total	344	
*FY*01W.01*	T	G	C	T	G	C	G	C	C	G	C	G	G	G	C	1	4869
*FY*02W.01*	T	A	C	T	A	C	G	C	C	G	C	G	G	G	C	2	9640 - 14512
															Sub-total	3	
															Total	902	

* Variant positions in the 1 intron and synonymous variants in the 2 exons are not listed.
† SNV does not affect expression of Fy antigens.[1,2]
‡ SNV has no known effect on Fy antigens.
-67T>C, *GATA box* mutation[3]; 125G>A, Fy^a/Fy^b polymorphism[4]; 265C>T, associated with $Fy(b+^w)$, Fy^x phenotype[2]; 977C>T, associated with $Fy(b+^w)$ phenotype (ISBT; indicated as $Fy(a+^w)$ by Schoeman et al.[5]).
Nucleotide positions are shown according to the human reference sequence (NG_011626.3) and defined using the first nucleotide of the coding sequence (CDS) of the NM_002036.2 isoform as nucleotide position 1.

References

1. Tournamille C, Colin Y, Cartron JP, et al. Disruption of a GATA motif in the Duffy gene promoter abolishes erythroid gene expression in Duffy-negative individuals. Nat Genet 1995;10:224-8.
2. Tournamille C, Le Van Kim C, Gane P, et al. Molecular basis and PCR-DNA typing of the Fya/fyb blood group polymorphism. Hum Genet 1995;95:407-10.
3. Olsson ML, Smythe JS, Hansson C, et al. The Fy(x) phenotype is associated with a missense mutation in the Fy(b) allele predicting Arg89Cys in the Duffy glycoprotein. Br J Haematol 1998;103:1184-91.
4. Schoeman EM, Roulis EV, Liew YW, et al. Targeted exome sequencing defines novel and rare variants in complex blood group serology cases for a red blood cell reference laboratory setting. Transfusion 2018;58:284-93.
5. Jongruamklang P, Gassner C, Meyer S, et al. Matrix-assisted laser desorption/ionization time-of-flight mass spectrometry analysis of 36 blood group alleles among 396 Thai samples reveals region-specific variants. Transfusion 2018;58:1752-62.
